# 3D TractFormer: 3D Direct Volumetric White Matter Tract Segmentation with Hybrid Channel-Wise Transformer

**DOI:** 10.3390/s26031068

**Published:** 2026-02-06

**Authors:** Xiang Gao, Hui Tian, Xuefei Yin, Alan Wee-Chung Liew

**Affiliations:** School of Information & Communication Technology, Griffith University, Gold Coast, QLD 4215, Australia; x.gao@griffith.edu.au (X.G.); x.yin@griffith.edu.au (X.Y.); a.liew@griffith.edu.au (A.W.-C.L.)

**Keywords:** white matter tract, 3D segmentation, diffusion MRI

## Abstract

Segmenting white matter tracts in diffusion-weighted magnetic resonance imaging (dMRI) is of vital importance for brain health analysis. It remains a challenging task due to the intersection and overlap of tracts (i.e., multiple tracts coexist in one voxel) and the data complexity of dMRI images (e.g., 4D high spatial resolution). Existing methods that demonstrate good performance implement direct volumetric tract segmentation by performing on individual 2D slices. However, this ignores 3D contextual information, requires additional post-processing, and struggles with the boundary handling of 3D volumes. Therefore, in this paper, we propose an efficient 3D direct volumetric segmentation method for segmenting white matter tracts. It has three key innovations. First, we propose to deeply interleave convolutions and transformer blocks into a U-shaped network, which effectively integrates their respective strengths to extract spatial contextual features and global long-distance dependencies for enhanced feature extraction. Second, we propose a novel channel-wise transformer, which integrates depth-wise separable convolution and compressed contextual feature-based channel-wise attention, effectively addressing the memory and computational challenges of 4D computing. Moreover, it helps to model global dependencies of contextual features and ensures each hierarchical layer focuses on complementary features. Third, we propose to train a fully symmetric network with gradually sized volumetric patches, which can solve the challenge of few 3D training samples and further reduce memory and computational costs. Experimental results on the largest publicly available tract-specific tractograms dataset demonstrate the superiority of the proposed method over the current state-of-the-art methods.

## 1. Introduction

White matter tract segmentation in diffusion-weighted magnetic resonance imaging (dMRI) is a critical component in the study of brain function and the diagnosis of diseases [1,2]. It presents significant challenges due to the complex nature of white matter tracts and dMRI data, such as the presence of intersecting and overlapping tracts within a single voxel and the 4D high spatial resolution. Various methods have been proposed for automated white matter tract segmentation [3,4,5], which can be broadly classified into two categories: (1) regions-of-interest (ROI)/clustering-based methods, and (2) direct volumetric segmentation.

ROI/clustering-based methods achieve tract segmentation by grouping the fiber streamlines into anatomically defined fiber tracts. They typically require an entire series of processing steps, including atlas registration, tractography, and clustering or parcellation. Therefore, these methods are computationally expensive and tedious for fine-tuning. Direct volumetric segmentation generates complete tract segmentation directly from the input images. It does not require performing tractography that is sensitive to the choice of tracking algorithm and is therefore simple and efficient.

In the early stage, many efforts have been made on direct volumetric segmentation by using techniques such as Markov random field and *k*-nearest neighbors [6,7,8]. However, the segmentation quality achieved by these methods is limited, which in turn has forced the subsequent research to focus on ROI/clustering-based methods. Direct volumetric segmentation of white matter tracts has achieved promising performance until recently, when Wasserthal et al. [4] proposed using a Unet-based network, TractSeg, to segment white matter tracts in the field of fiber orientation distribution function (fODF) peaks [9].

After that, several studies on enhancing direct volumetric tract segmentation have been conducted, as reviewed and discussed in [5,8]. However, these studies are based on 2D slices. They ignore 3D contextual information, which may lead to inconsistency and discontinuity in segmentation of 3D volumes. Furthermore, they require additional post-processing steps and encounter difficulties in handling the boundaries of 3D volumes. This motivates us to develop an efficient 3D direct volumetric segmentation method for segmenting white matter tracts.

In the literature, Wasserthal et al. [10] attempted to employ a 3D Unet for direct volumetric tract segmentation but failed. Li et al. [11] achieved 3D direct volumetric tract segmentation in native diffusion space, but their method only worked for six gradient directions and single tract segmentation. Nelkenbaum et al. [12] implemented 3D direct volume tract segmentation in MRI images, where the data has very different characteristics from dMRI data. This shows that the main challenges in developing 3D direct volumetric tract segmentation in dMRI are: (1) the inadequacy of relying solely on 3D convolutions to learn the features required for effective segmentation from high angular resolution data, (2) the large memory and computational cost associated with 4D computations impacting the development of sophisticated networks, and (3) the scarcity of 3D annotation data affecting the network training.

In this paper, we address the above challenges through three major innovative designs and, for the first time, propose an efficient 3D direct volumetric white matter tract segmentation method that can simultaneously generate segmentation of 72 anatomically well-described tracts. We name the proposed method 3D TractFormer. It has the following key contributions.

- We propose to deeply interleave convolutions and transformer blocks into a U-shaped network. This effectively integrates the respective strengths of both to extract spatial contextual features and global long-distance dependencies, thereby enhancing feature extraction for direct volumetric tract segmentation.
- We propose a novel memory- and computationally efficient channel-wise transformer. It integrates depth-wise separable convolution and compressed contextual feature-based channel-wise attention. It can effectively address the memory and computational challenges of 4D computation. Moreover, it facilitates the modeling of global dependencies of contextual features and ensures that each hierarchical layer of the 3D TractFormer focuses on complementary features for tract segmentation.
- We propose to train a fully symmetric network with gradually sized volumetric patches, which can solve the challenge of few 3D training samples and further reduce memory and computational costs.
- We show experimentally that the proposed 3D TractFormer outperforms existing methods that have demonstrated state-of-the-art performance for white matter tract segmentation.

## 2. Related Works

### 2.1. White Matter Tract Segmentation

White matter tract segmentation in dMRI refers to delineating streamlines/voxels of the anatomical white matter tracts in brain [13,14]. In the context of direct volumetric tract segmentation, which is the focus of this paper, promising segmentation performance was first achieved when Wasserthal et al. [4] proposed using a deep network to achieve segmentation in the field of fODF peaks. Specifically, they proposed using dMRI-derived fODF peaks as a compact representation of raw dMRI data and as the input for direct volumetric tract segmentation. This solves the problem of large channel size of the input caused by high angular resolution data and makes the segmentation method independent of dMRI acquisition settings. This also facilitates the application of deep learning methods for direct volumetric tract segmentation. This work stands as a significant milestone and has become the benchmark in the field.

Since then, several studies on enhancing direct volumetric tract segmentation have been proposed based on this idea, as reviewed and discussed in [5,8]. Among them, several methods have been proposed to improve network architecture to enhance the segmentation performance [10,15]. For example, Dong et al. [15] enhanced TractSeg by introducing dual inputs, resulting in improved segmentation. Several methods have been proposed to achieve direct volumetric tract segmentation with few training samples or in low quality/resolution images [14,16,17]. A systematical investigation of a large variety of methods for direct volumetric tract segmentation is provided in a most recent study [18]. However, these methods are based on 2D slices, which ignore 3D contextual information, require extra post-processing, and encounter difficulties in handling the edges of 3D volumes.

This motivates us to develop an efficient 3D direct volumetric tract segmentation method. In the literature, Wasserthal et al. [10] attempted to utilize a 3D Unet for direct volumetric tract segmentation but failed. Li et al. [11] achieved 3D direct volumetric tract segmentation in native diffusion space; however, their method was only effective for six gradient directions and single tract segmentation. Nelkenbaum et al. [12] implemented 3D direct volume tract segmentation in MRI images, which have significantly different characteristics from dMRI data. These highlight the primary challenges in developing 3D direct volumetric tract segmentation: (1) the insufficiency of relying solely on 3D convolutions to learn the necessary features for effective segmentation from high angular resolution data, (2) the substantial computational demands of 4D computations impacting the development of sophisticated networks, and (3) the scarcity of 3D annotation data affecting the network training. In this paper, we carefully design a 3D TractFormer to address these challenges.

### 2.2. Vision Transformer

The transformer [19] has strong capability to learn long-range dependencies due to its core component self-attention. It has been used in various vision tasks such as recognition [20], segmentation [21], and restoration [22], as discussed in [23,24]. Most current vision transformers apply self-attention in the spatial dimension and model the dependencies between pixels. Since the computational complexity of self-attention grows quadratically with spatial resolution, these methods usually apply self-attentions within small windows or patches. This is not conducive to capturing true global long-range dependencies, especially on high-resolution images. Channel-wise transformers have been proposed to solve this limitation to a certain extent [25,26]. However, when they are directly used to compute attention for 3D images, the computational complexity is still very high, as it increases cubically with volume dimension. Therefore, in this paper, we propose a new memory- and computationally efficient channel-wise transformer for 3D attention computation. It can model global long-range dependencies of contextual features while maintaining memory and computational efficiency.

More recently, the medical image segmentation community has actively explored more efficient global context modeling for volumetric data. For example, MA-SAM adapts the Segment Anything Model to 3D medical image segmentation via modality-agnostic volumetric adapters [27]. Slim UNETR further investigates efficient hybrid transformer designs for 3D segmentation under limited computational resources [28]. In addition, state space model-based architectures have emerged as an alternative direction for long-range modeling with improved efficiency, including SegMamba for 3D volumetric segmentation [29] and VM-UNet for medical image segmentation [30]. These recent studies reinforce the importance of designing memory-efficient mechanisms to capture global dependencies in 3D segmentation tasks.

### 2.3. Hybrid Convolution and Transformer

Transformers excel in capturing distant dependencies, while convolution networks excel in extracting spatial context [23,24]. As analyzed above, exploiting their complementarity in feature learning is beneficial for 3D direct volumetric tract segmentation. Most existing hybrid convolution and transformer methods involve shallow block-level blending. For example, in [21,31], transformer blocks are employed in the encoder path for feature encoding, while convolution blocks are adopted in the decoder path for feature decoding. In [32], transformer blocks are only integrated into skip-connection layers. In [33], convolutions and transformer blocks are interleaved in block-level. In [22,25], convolutions are blended in transformers, but are not blended with transformer blocks. Therefore, inspired by these works, in this paper, we propose a deep hybrid mode, where convolutions and transformers are interleaved both at block-level and within the attention computation.

Recent work has also continued to advance hybrid segmentation architectures that balance local inductive bias, global dependency modeling, and efficiency. For 3D segmentation, LW-CTrans proposes a lightweight CNN and transformer hybrid network [34], and Switch-UMamba integrates convolution with state space modeling for medical image segmentation [35]. Beyond volumetric segmentation, recent U-Net enhancements for related dense prediction tasks also emphasize dynamic multi-scale fusion and attention-based feature interaction, such as APU-Net for polyp segmentation [36]. These developments provide useful context for positioning our design choices in a broader segmentation landscape.

## 3. Methods

We propose 3D TractFormer, a hybrid channel-wise transformer for efficient 3D direct volumetric white matter tract segmentation. The overall pipeline is first presented in Section 3.1. Then, the two core components, multi-head convolution-based channel-wise attention (MCCA) and context-enhanced feed-forward network (CFFN) of the proposed channel-wise transformer, are detailed in Section 3.2 and Section 3.3, respectively. Finally, we provide the network configuration and training strategy in Section 3.4.

### 3.1. Overall Pipeline

**Figure 1:**
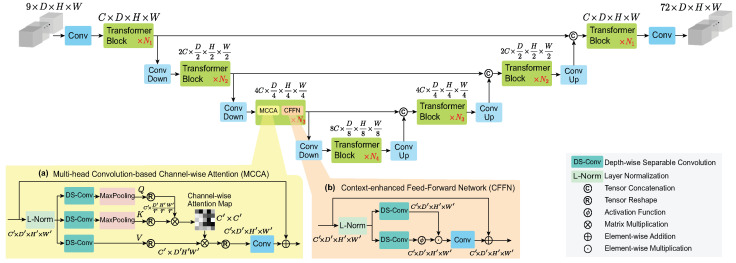
Architecture of the proposed 3D TractFormer. It employs a U-shaped encoder–decoder framework in which convolutions (blue rectangles) and transformer blocks (purple rectangles) are interleaved hierarchically at multiple scales. The proposed channel-wise transformer has two key components: (**a**) multi-head convolution-based channel-wise attention (MCCA) that models global spatial contextual feature dependence across channels, and (**b**) a context-enhanced feed-forward network (CFFN) that facilitates the propagation of important contextual information.

Figure 1 illustrates the architecture of 3D TractFormer. Its input is a 3D fiber orientation map with H×W×D×9 voxels. As mentioned above, instead of using raw dMRI image values as input, we use fiber orientation peaks as input for direct volumetric tract segmentation. This reduces the number of input channels from 270 to 9, significantly reducing the memory and computational cost. Fiber orientation peaks are computed from dMRI image values using the method in [4]. These 9 channels correspond to the three anatomically defined principal fiber directions, and each principal direction has three input channels. The output is the complete segmentation for 72 white matter tracts, which is concatenated channel-wise into a segmentation mask of H×W×D×72 voxels. This enables multi-label segmentation where multiple tracts coexist in a voxel.

3D TractFormer has a U-shaped encoder–decoder framework, where convolutions (including conv, conv down, and conv up) and transformer blocks are interleaved hierarchically in multiple scales. This design facilitates the extraction of complementary spatial contextual features and global dependencies, which is beneficial for direct volumetric tract segmentation as analyzed above. The first convolution encodes spatial contextual 3D features, while the last convolution generates the segmentation from decoded features. The down convolutions are responsible for encoding spatial contextual features while halving their spatial resolution and doubling their channels, and vice versa for the up convolutions. Skip-connections [37] are adopted to facilitate feature decoding. The network is symmetrical, with a 4-layer encoder–decoder structure. Each layer contains multiple transformer blocks, whose numbers are gradually increased to maintain efficiency. The transformer blocks capture global contextual long-range dependencies through its two core components MCCA and CFFN, as detailed below.

### 3.2. Multi-Head Convolution-Based Channel-Wise Attention

The main computational cost in a transformer comes from its self-attention computation. In a conventional transformer, the computational complexity of the key query dot product is $O(H^{2}W^{2}D^{2})$ for an input of size H×W×D. By using a vanilla channel-wise transformer, the computational complexity can be reduced to O(HWD). However, this is still computationally expensive, as a typical size of H/W/D can result in a very large HWD and this computational complexity will increase cubically with the volumetric dimension. To alleviate this problem, we propose MCCA (Figure 1a), which has a computational complexity of $O(HWD/r^{3})$.

Another core design of MCCA is to use depth-wise separable convolution instead of the learnable weight matrices in conventional transformers to generate the query Q, key K, and value V. Given the small number of parameters in depth-wise separable convolution, this does not introduce much computational complexity, but offers two distinct advantages. First, it compensates for the limitations of transformers in modeling spatial contextual information by introducing a convolution operation. Second, it enables us to model global dependencies of spatial contextual features, which offers unique advantages for direct volumetric tract segmentation.

Specifically, for an input feature $F\in R^{H^{\prime }\times W^{\prime }\times D^{\prime }\times C^{\prime }}$, our MCCA first generates its $Q\in R^{\frac{H^{\prime }}{r}\times \frac{W^{\prime }}{r}\times \frac{D^{\prime }}{r}\times C^{\prime }}$ and $K\in R^{\frac{H^{\prime }}{r}\times \frac{W^{\prime }}{r}\times \frac{D^{\prime }}{r}\times C^{\prime }}$ by $Q=f_{m}\left(f_{ds}^{Q}\left(F\right)\right)$ and $K=f_{m}\left(f_{ds}^{K}\left(F\right)\right)$, respectively, where $f_{ds}^{\left(\cdot \right)}$ is the depth-wise separable convolution and $f_{m}$ is the max-pooling. We use a pooling factor of *r* to reduce the dimension of the extracted spatial contextual feature maps, which can reduce the complexity of the attention computation by a factor of $r^{3}$. Then, the Q and V are reshaped to make spatial contextual features in each channel as tokens to compute channel-wise attention. Thus, the channel-wise attention map is of size $C^{\prime }\times C^{\prime }$. We generate $V\in R^{H^{\prime }\times W^{\prime }\times D^{\prime }\times C^{\prime }}$ by $V=f_{ds}^{V}\left(F\right)$ and also reshape it along the channel. Overall, our MCCA can be formulated as:

(1) $$\begin{array}{cc} \; & \mathrm{Attention}\left(Q^{\prime },K^{\prime },V^{\prime }\right)=\mathrm{Softmax}\left(\frac{Q^{\prime }{K\prime }^{T}}{\alpha }\right)V^{\prime }\\ \; & F^{\prime }=f_{c}^{1}\left(\mathrm{Attention}\left(Q^{\prime },K^{\prime },V^{\prime }\right)\right)+F, \end{array}$$

where $Q^{\prime }\in R^{C^{\prime }\times \frac{H^{\prime }}{r}\frac{W^{\prime }}{r}\frac{D^{\prime }}{r}}$, $K^{\prime }\in R^{C^{\prime }\times \frac{H^{\prime }}{r}\frac{W^{\prime }}{r}\frac{D^{\prime }}{r}}$, and $V^{\prime }\in R^{C^{\prime }\times H^{\prime }W^{\prime }D^{\prime }}$ are the reshaped tensors. α is a learnable parameter used to control the magnitude of the dot product, $f_{c}^{1}$ is a 1×1×1 convolution used to refine features, and $F^{\prime }$ is the output feature generated by MCCA. Similar to the conventional multi-head self-attention [22], we partition the channel number into the heads and simultaneously learn separate attention maps.

### 3.3. Context-Enhanced Feed-Forward Network

The feed-forward network in the conventional transformer uses only two 1×1 convolutions and a non-linear operation between them to transform features. Thus, its capability to exploit spatial contextual information is limited. However, spatial contextual information is important for white matter tract segmentation, as evidenced by the much-studied convolution-based methods. To address this limitation, we propose CFFN (Figure 1b), which integrates depth-wise separable convolutions into the conventional feed-forward network to emphasize spatial context. Also, it incorporates a gate mechanism as in [25] to further control the features passed forward to the next layer. This enables different layers in the hierarchical 3D TractFormer to focus on complementary features, ultimately enhancing feature learning.

Overall, our CFFN can be formulated as:

(2) $$\begin{array}{cc} \; & F^{\prime \prime }=f_{c}^{2}\left(f_{ds}^{a}\left(F^{\prime }\right)⊙\phi \left(f_{ds}^{b}\left(F^{\prime }\right)\right)\right)+F^{\prime }, \end{array}$$

where $F^{\prime }$ is the output from MCCA, $f_{ds}^{a}$ and $f_{ds}^{b}$ are two depth-wise separable convolutions, ϕ is the GELU activation function [38], ⊙ denotes the element-wise multiplication, $f_{c}^{2}$ is a 1×1×1 convolution used to refine features, and $F^{\prime \prime }$ is the output feature generated by CFFN.

MCCA leverages spatial contextual features to enhance the encoding of global dependencies, while CFFN further enriches these features using contextual information and controls features forward to the next layer, ensuring that different hierarchical layers focus on complementary information. Their synergy empowers 3D TractFormer to effectively extract features that facilitate white matter tract segmentation.

### 3.4. Network Configuration and Training Strategy

Our 3D TractFormer has four hierarchical levels; from the first to the fourth level, the numbers of transformer blocks are [1, 2, 4, 8], the numbers of attention heads in MCCA are [1, 2, 4, 8], and the numbers of channels are [36, 72, 144, 288]. The pooling factor in MCCA is r=2. The convolutions have a kernel size of 3×3×3. The down and up convolutions have a kernel size of 4×4×4 with a stride of 2. The depth-wise separable convolutions have kernel sizes of 3×3×3 and 1×1×1. The final layer incorporates the sigmoid activation function to transform output values into probabilities. A threshold of 0.5 is applied to distinguish background voxels (assigned 0) and foreground voxels (assigned 1). To train the network, binary cross-entropy loss is computed between the generated segmentation masks and the ground-truth masks.

In 3D direct volumetric white matter tract segmentation, the computational complexity can increase quartically with the dimension (i.e., H/W/D/C). To improve computation efficiency, we propose to train 3D TractFormer with small volumetric patches. This also naturally increases the amount of training data and reduces the requirement for the number of annotations. Our 3D TractFormer is symmetric, so it can be trained with small patches but tested on large images. That is, at test time, we directly perform whole volume inference on the complete 3D sample and do not use any patch merging procedure. To ensure its performance on images of different sizes, we also incorporate progressively larger patches for training. This is somewhat similar to the ideas of progressive learning [39] and curriculum learning [40]. With this training strategy, we partially solve the challenges of high memory and computational cost and lacking sufficient annotations for training in 3D direct volumetric white matter tract segmentation.

## 4. Experimental Results

### 4.1. Datasets

We use the largest publicly available tract-specific tractograms dataset provided in [4] for white matter tract segmentation. It contains tractogram annotations for 72 anatomically well-defined white matter tracts across 105 subjects in the WU-Minn Human Connectome Project (HCP) [2]. These annotations are used as ground truth. The fiber orientation peaks input to the 3D TractFormer are computed from the corresponding dMRI scans in the HCP dataset. These dMRI scans have 270 diffusion gradients and a spatial resolution of 145×174×145. We crop them to 144×144×144 for experiments, without loss of any brain tissue.

### 4.2. Experimental Settings

We use the AdamW optimizer [41] with $\beta_{1}=0.9,\;\beta_{2}=0.999$, and weight decay of $1\times 10^{-4}$ to train the 3D TractFormer. The initial learning rate is set to be $3\times 10^{-4}$, which is gradually reduced to $1\times 10^{-6}$ through the cosine annealing algorithm [42]. We train 3D TractFormer for 200 epochs, with each epoch comprising 1000 iterations. The volumetric patches used for training have sizes 64×64×64 and 80×80×80, with batch sizes 6 and 2, respectively. The experiments were carried out utilizing PyTorch (version 2.4.0). and executed on a cluster of four Nvidia V100 GPUs (Nvidia, Santa Clara, CA, USA), each equipped with 32 GB of memory.

Five-fold cross-validation is used, where each fold contains 63 training subjects, 21 validation subjects, and 21 test subjects. The model that achieved the highest performance on the validation set is used for the final evaluation on the test set.

### 4.3. Evaluation Metrics

We employ the Dice similarity coefficient (DSC) and the relative volume difference (RVD) as quantitative metrics to evaluate the segmentation performance. The DSC is a widely recognized overlap-based segmentation metric, while the RVD is a prominent size-based segmentation metric. They complement each other, offering a comprehensive evaluation of our 3D TractFormer. Additionally, we employ a two-tailed Wilcoxon signed-rank test with α=0.05 [43] to evaluate statistical significance. For each pair of methods, we perform a paired test at the tract level by comparing the DSCs (and RVDs) for each of the 72 tracts across all 105 subjects, yielding 72×105=7560 paired differences for one comparison. The null hypothesis is that the median of the paired differences is zero, and the alternative hypothesis is that it is nonzero. In Table 1, the reported *p* values compare each method with the method listed immediately above it within the same block, namely within the 2D slice-based block and within the 3D volumetric block. For Table 4, the reported *p* values are computed by comparing each ablation variant against the full 3D TractFormer.

### 4.4. Performance Evaluation

#### 4.4.1. Comparison with State-of-the-Art Methods

We compare our 3D TractFormer with the TractSeg method [10], which is the current benchmark in the field. We also compare it with a variety of state-of-the-art methods that have been applied to white matter tract segmentation for all 105 subjects in the HCP. These methods include the UNet++ [45], DS-UNet [10], UNet3+ [46], and Attention UNet [44], as investigated in the latest study [18], as well as the latest method AC-UNet that was specially designed for WMT segmentation [47]. Since most of these baselines operate on 2D slices, we additionally incorporate volumetric 3D baselines to provide a more comprehensive comparison. Concretely, we adapt Neuro4Neuro [11], a direct 3D tract segmentation approach in native diffusion space, to our setting and denote this variant as m-Neuro4Neuro. We also include two additional 3D methods, namely nnUNet [48] and Uformer [11], to benchmark against strong volumetric segmentation pipelines. Collectively, these baselines cover both slice-based and fully 3D formulations, enabling a thorough evaluation of our method.

Table 1 compares the quantitative evaluation results of these methods. It includes the mean and standard deviation (STD) of Dice scores and RVD values for each method, which are computed over the five folds between the generated segmentation and the ground truth. Note that higher Dice scores indicate better performance, while lower RVD values indicate higher performance. Additionally, Table 1 reports *p* values from paired Wilcoxon signed-rank tests, where each method is compared with the method listed immediately above it within the same block, namely within the 2D block and within the 3D block. As can be seen, our 3D TractFormer outperforms the other methods, showcasing the best segmentation performance and statistically significant improvements in both Dice scores and RVD values. This demonstrates its superiority in 3D direct volumetric tract segmentation. The performance achieved by m-Neuro4Neuro is obviously lower than other methods, which is explainable because Neuro4Neuro was originally designed for single tract segmentation in native diffusion space. This also underscores the importance of developing tailored methods for 3D white matter tract segmentation.

**Figure 2:**
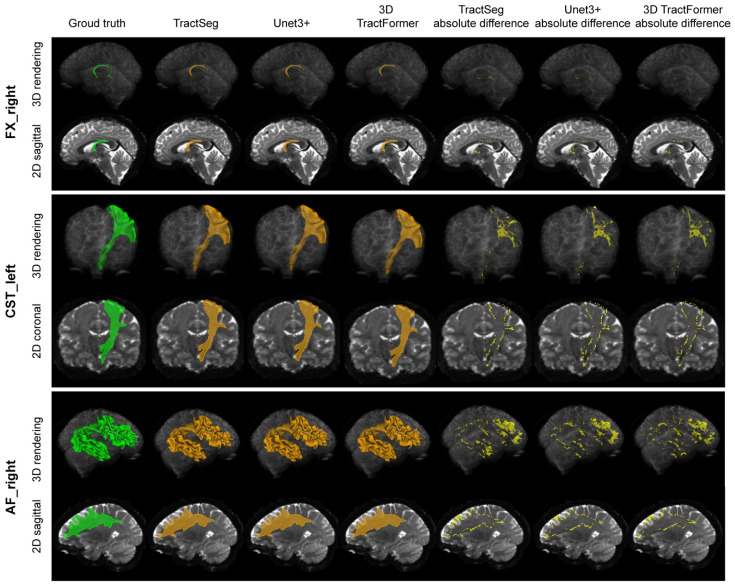
Visual comparison of segmentation results of our 3D TractFormer, TractSeg, and Unet3+ for small-scale (FX_right), medium-scale (CST_left), and large-scale (AF_right) white matter tracts in terms of 3D renderings and 2D cross-sectional views. Green indicates the ground truth, orange indicates the predicted segmentation, and yellow indicates the absolute difference from the ground truth.

Figure 2 provides a qualitative evaluation of our segmentation results in comparison to those of TractSeg and Unet3+. Both the cross-sectional views and 3D renderings of the segmentation results are illustrated. For better observation, we also present the absolute differences between segmentation results and the manual delineations. It can be seen that our results exhibit greater similarity to the manual delineations compared to those of TractSeg and Unet3+, with smaller absolute differences. This further demonstrates the superiority of 3D TractFormer.

#### 4.4.2. Effectiveness on Small-Scale and Challenging Tracts

**Figure 3:**
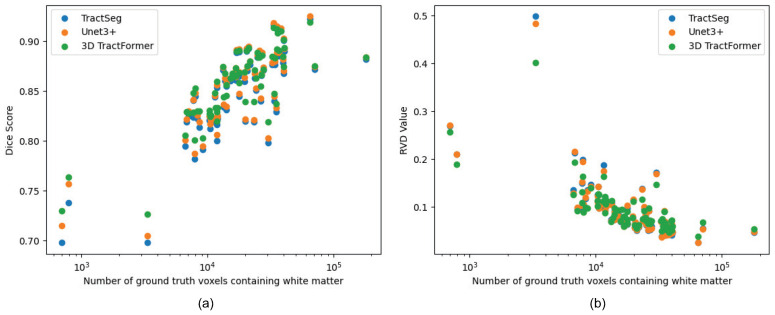
Graph depicting the relationship between tract size and segmentation performance across 72 white matter tracts. (**a**) Mean Dice score and (**b**) mean RVD value. The dots indicate white matter tracts.

Small-scale tracts are inherently more difficult to segment, and slight changes in segmentation volume can lead to relatively large impacts on their performance. For a more in-depth evaluation of 3D TractFormer, we compare its segmentation performance with those of TractSeg and Unet3++ (the current top-performing method in the literature, as shown in Table 1) on varied tract sizes. Figure 3 plots the tract size versus segmentation performance in terms of Dice score and RVD value for 72 white matter tracts. As can be seen, 3D TractFormer achieves better results on small-scale tracts. This supports our claim that direct 3D segmentation can leverage 3D spatial context to capture information of small volume, thereby improving segmentation performance.

Among the 72 tracts, some are more challenging to segment than others. Liu et al. [14] have identified seven particularly challenging tracts that exhibit narrow or uncinate shapes. To evaluate the performance of our 3D TractFormer in tough scenarios, we compare the segmentation performance of our 3D TractFormer and Unet3+ on each of these challenging tracts. Table 2 presents the quantitative evaluation results. As can be seen, 3D TractFormer achieves consistently higher Dice scores for all these tracts, demonstrating its effectiveness in difficult scenarios.

#### 4.4.3. Model Complexity and Computational Cost

We further quantify the efficiency of our method by reporting model complexity in Table 3, including the number of trainable parameters, TMACs, and TFLOPs. For a fair comparison, TMACs and TFLOPs are computed for a single inference pass using the same input resolution of 9×144×144×144. As shown in Table 3, 3D TractFormer achieves a favorable tradeoff between accuracy and efficiency, requiring only 8.84 M parameters and 0.431 TMACs, which is substantially lower than the transformer-based 3D baseline Uformer. Compared with nnUNet, 3D TractFormer has comparable parameter scale and lower computational cost, while delivering better segmentation performance as shown in Table 1.

### 4.5. Ablation Study

We conduct ablation studies to quantify the contributions of the two key components in the proposed channel-wise transformer, namely MCCA and CFFN. All ablation models share the same overall 3D TractFormer backbone, training protocol, and evaluation settings as the full model, differing only in the corresponding module replacement. 3D TractFormer-xMCCA removes multi-head convolution-based channel-wise attention, weakening global dependency modeling from spatial context. 3D TractFormer-xCFFN removes context-enhanced transformation and gating, reducing spatial context enrichment and selective feature propagation across stages.

Table 4 reports Dice and RVD. The full 3D TractFormer achieves the best performance with Dice 0.857(±0.003) and RVD 0.096(±0.005). Replacing CFFN yields a consistent degradation, with Dice decreasing to 0.853(±0.003) and RVD increasing to 0.097(±0.005). Replacing MCCA leads to a larger performance drop, with Dice further decreasing to 0.852(±0.003) and RVD increasing to 0.097(±0.005). The *p* values, computed against 3D TractFormer, indicate that both degradations are statistically significant. Overall, these results verify that MCCA and CFFN are both necessary. MCCA plays a dominant role in improving feature learning, while CFFN provides additional gains by enhancing spatial context modeling and controlling feature propagation, and their combination yields the strongest volumetric tract segmentation performance.

## 5. Conclusions

This paper proposed 3D TractFormer, which solves three challenges in 3D direct volumetric tract segmentation and achieves state-of-the-art segmentation performance. Its success relies on its three main innovations: (1) the deep blending of convolution and converter blocks in the U-shaped network effectively enhances complementary feature extraction, (2) the memory- and computationally efficient channel-wise transformer facilitates the modeling of global dependencies of contextual features, and (3) the efficient 3D TractFormer training with gradually sized volumetric patches.

## Figures and Tables

**Table 1:** Comparison of segmentation performance of our 3D TractFormer and the state-of-the-art methods in terms of mean and STD Dice scores and RVD values. *p* values (α=0.05) are obtained using a two-tailed Wilcoxon signed-rank test and are computed against the method listed immediately above within the same block, that is, within the 2D block and within the 3D block. N/A indicates that no reference method is defined for that row. Bold values indicate the best performance under the corresponding metric.

Methods	2D/3D	Dice Scores (↑)		RVD Values (↓)	
		Mean (STD)	*p*-Value	Mean (STD)	*p*-Value
TractSeg [4]	2D	0.849 (±0.003)	N/A	0.097 (±0.006)	N/A
Attention Unet [44]		0.850 (±0.003)	<0.000	0.097 (±0.005)	0.599
DS-Unet [10]		0.851 (±0.002)	0.008	0.097 (±0.005)	0.564
Unet++ [45]		0.852 (±0.003)	<0.000	**0.096** (±0.005)	<0.000
Unet3+ [46]		0.853 (±0.003)	<0.000	0.097 (±0.005)	0.001
AC-UNet [47]		0.856 (±0.003)	<0.000	0.096 (±0.005)	0.001
nnUNet [48]	3D	0.842 (±0.040)	N/A	0.109 (±0.006)	N/A
m-Neuro4Neuro [11]		0.844 (±0.004)	<0.000	0.101 (±0.006)	<0.000
Uformer [11]		0.852 (±0.003)	<0.000	0.097 (±0.005)	<0.000
3D TractFormer		**0.857** (±0.003)	<0.000	**0.096** (±0.005)	<0.000

**Table 2:** Comparison of segmentation performance in terms of mean and STD Dice scores and RVD values of 3D TractFormer versus Unet3+ and AC-Unet on the seven most challenging tracts. Bold values indicate the best performance for each tract under the corresponding metric.

Tracts	Mean Dice Scores (↑)			Mean RVD Values (↓)		
	Unet3+	AC-UNet	3D TractFormer	Unet3+	AC-UNet	3D TractFormer
CA	0.704	0.706	**0.726**	0.482	0.482	**0.402**
FX_left	0.757	0.757	**0.763**	0.210	0.210	**0.189**
FX_right	0.715	0.714	**0.730**	0.271	0.271	**0.256**
ILF_left	0.822	0.823	**0.829**	0.105	**0.104**	0.106
ILF_right	0.806	0.808	**0.819**	**0.115**	**0.115**	0.121
UF_left	0.787	0.789	**0.801**	0.195	0.194	**0.163**
UF_right	0.819	0.820	**0.829**	0.133	0.133	**0.098**

**Table 3:** Comparison of model complexity measured by parameters (M), TMACs, and TFLOPs.

Methods	Parameters (M)	TMACs	TFLOPs
TractSeg [4] (2D)	9.24	0.696	1.395
Attention Unet [44] (2D)	34.89	3.05	6.11
DS-Unet [10] (2D)	55.505	4.296	8.605
Unet++ [45] (2D)	9.05	1.369	2.742
Unet3+ [46] (2D)	27.179	8.848	17.695
AC-UNet [47] (2D)	22.03	17.26	34.86
nnUNet [48] (3D)	4.09	0.69	1.39
m-Neuro4Neuro [11] (3D)	51.79	2.55	5.1
Uformer [11] (3D)	47.45	0.672	1.361
3D TractFormer (3D)	8.84	0.431	0.867

**Table 4:** Ablation study of 3D TractFormer. 3D TractFormer-xMCCA replaces MCCA with a simple MLP, and 3D TractFormer-xCFFN replaces CFFN with a simple MLP. Results are reported as mean (STD) for Dice and RVD. *p* values (α=0.05) are obtained using a two-tailed Wilcoxon signed-rank test and are computed by comparing each ablation variant against the full 3D TractFormer. Bold values indicate the best performance under the corresponding metric. 3D TractFormer serves as the reference (full) model for statistical comparison.

Methods	Dice Scores		RVD Values	
	Mean (STD)	*p*-Value	Mean (STD)	*p*-Value
3D TractFormer	**0.857** (±0.003)	N/A	**0.096** (±0.005)	N/A
3D TractFormer-xCFFN	0.853 (±0.003)	<0.000	0.097 (±0.005)	<0.000
3D TractFormer-xMCCA	0.852 (±0.003)	<0.000	0.097 (±0.005)	<0.000

## Data Availability

The original contributions presented in this study are included in the article. Further inquiries can be directed to the corresponding author.
